# JC Polyomavirus (JCV) and Monoclonal Antibodies: Friends or Potential Foes?

**DOI:** 10.1155/2013/967581

**Published:** 2013-06-25

**Authors:** Roberta Antonia Diotti, Akira Nakanishi, Nicola Clementi, Nicasio Mancini, Elena Criscuolo, Laura Solforosi, Massimo Clementi

**Affiliations:** ^1^Microbiology and Virology Institute, Vita-Salute San Raffaele University, Via Olgettina 58, 20132 Milan, Italy; ^2^Department of Aging Intervention, National Center for Geriatrics and Gerontology, 35 Gengo, Morioka, Obu, Aichi 474-8522, Japan

## Abstract

Progressive multifocal leukoencephalopathy (PML) is a demyelinating disease of the central nervous system (CNS), observed in immunodeficient patients and caused by JC virus ((JCV), also called JC polyomavirus (JCPyV)). After the HIV pandemic and the introduction of immunomodulatory therapy, the PML incidence significantly increased. The correlation between the use of natalizumab, a drug used in multiple sclerosis (MS), and the PML development of particular relevance. The high incidence of PML in natalizumab-treated patients has highlighted the importance of two factors: the need of PML risk stratification among natalizumab-treated patients and the need of effective therapeutic options. In this review, we discuss these two needs under the light of the major viral models of PML etiopathogenesis.

## 1. Progressive Multifocal Leukoencephalopathy (PML) and JC Polyomavirus (JCV): An Epidemiological Overview

### 1.1. The Emergence of PML in the Monoclonal Antibody Era

Progressive multifocal leukoencephalopathy (PML) is a demyelinating disease of the central nervous system (CNS) usually observed in immunodeficient patients. The first case was described in 1958 [1], and the detection of inclusion bodies in the nuclei of damaged oligodendrocytes suggested a possible viral cause. The etiological agent of PML was isolated in 1971 and named JC virus ((JCV), also called JC polyomavirus, (JCPyV)), after the initials of the studied patient [2, 3].

After the HIV spread, the PML incidence has increased 50-fold compared to previous years and 80% of PML cases are represented by HIV-positive patients [4]. Since the advent of antiretroviral therapy, the incidence of PML in AIDS patients is still estimated to be 0.07/100 persons/year, and it has not decreased as significantly as other opportunistic infections [5–8].

In the very last years, PML has become a growing concern in other categories of patients, and its incidence remains high. The new cases of PML are associated to the use of novel immunomodulatory therapies in patients affected by several diseases, such as multiple sclerosis (MS), Crohn's disease, non-Hodgkin's lymphoma, systemic lupus erythematosus (SLE), rheumatoid arthritis (RA), and autoimmune hematological disorders [9, 10]. The incidence of PML in patients under immunomodulatory therapy depends on the drug used and on the treated disease. For example, the risk of PML during rituximab administration, an anti-CD20 humanized monoclonal antibody (mAb), has been estimated to be approximately 1/4000 when used in SLE patients and 1/25000 when used in RA [11]. An even higher incidence (1/500) was observed in psoriatic patients treated with efalizumab, a humanized mAb against a T lymphocytes adhesion molecule, and as a consequence efalizumab was voluntarily withdrawn from the market [12].

### 1.2. PML and Natalizumab: The Evidence

In the literature, the association between natalizumab administration and PML has been widely reported and described. Natalizumab is an IgG4/*κ* humanized mAb, which interferes with the interaction between *very late antigen-4* (VLA-4), expressed on leukocytes, and *vascular adhesion molecule-1 * VCAM-1 expressed on endothelial cells, thus preventing leukocyte extravasation in inflamed sites [13]. Natalizumab is generally well tolerated, but due to its correlation with PML, it was approved with a restricted distribution format in 2006. In particular, the risk of PML development during natalizumab treatment is very high, and it has been evaluated to be as high as 3.85 per 1000 patients [14], and survival rate is 70% (natalizumab-associated PML has improved survival rate compared with PML in other populations) [15].

Natalizumab is used in several autoimmune diseases but, in particular, for the MS treatment. MS is a chronic inflammatory autoimmune disease of the CNS affecting more than 2.5 million people worldwide, characterized by chronic leukocyte infiltration [16]. Most patients suffer from a relapsing-remitting course that is characterized by about one and two episodes of neurological deficits per year, that often tend to resolve, at least partly, after days to months [17, 18]. Natalizumab reduced the rate of clinical relapse at one year by 68% and the risk of sustained progression of disability by 42–54% over 2 years, turning out to be the most effective drug in MS treatment. Its efficacy in MS is probably correlated to its capacity of blocking leukocyte infiltration into the inflamed plaques within CNS [19].

**Figure 1:**
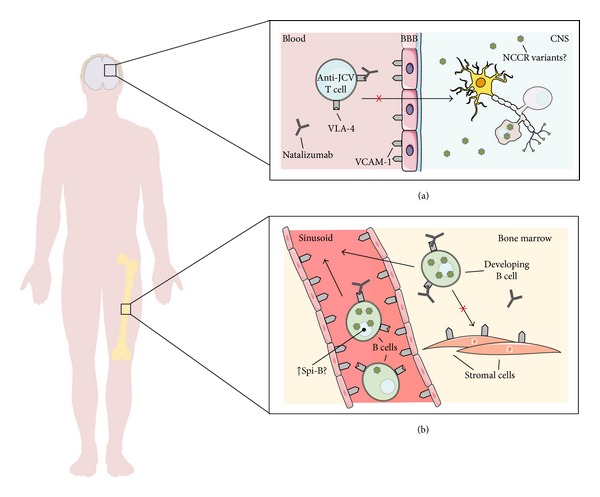
Development of progressive multifocal leukoencephalopathy during natalizumab treatment. This figure summarizes the three hypotheses on how natalizumab may lead to PML. (a) Natalizumab may prevent the entry of JCV-specific cytotoxic T cells into the brain, necessary for the control of latent JCV within infected oligodendrocytes. (b) Natalizumab inhibits the VLA-4-dependent homing and retentions of lymphocytes in bone marrow (sites of JCV latency), thus leading to an increase of JCV-infected peripheral leukocytes. Finally, another possible factor is the natalizumab-induced expression of Spi-B, a transcription factor that has been shown to increase JCV transcription.

On the other hand, the pathogenesis of PML in patients receiving natalizumab is complex, and it is not clear whether it is caused by a local (within CNS) or peripheral reactivation of JCV leading to a massive crossing of the blood-brain barrier (BBB) by free or B cells shuttled viral particles. To date, three main molecular mechanisms have been proposed. According to some authors, the blockage of VLA-4 by natalizumab may prevent the entry of JCV-specific cytotoxic T cells into the brain, necessary for the control of latent JCV within infected oligodendrocytes (the viral life cycle will be better explained in what follows) [20]. Another proposed possibility is that natalizumab may inhibit the VLA-4-dependent retention of lymphocytes in bone marrow and spleen (both sites of JCV latency), thus leading to an increase of JCV-infected peripheral leukocytes and to a possible increase of the peripheral JC viral load capable of crossing the BBB (this late aspect has not been confirmed, to date) [21]. Another suggested mechanism is the natalizumab-induced expression of factors involved in B-cell differentiation, such as transcription factor Spi-B, that has been shown to increase JCV transcription, thus probably leading to an increased viral load, at least *in vitro* (Figure 1) [22].

### 1.3. PML and Natalizumab: The Need of Risk Stratification

Natalizumab was first approved by Food and Drug Administration (FDA) for MS treatment in 2004. Due to the first confirmed cases of PML, its commercialization was suspended in March 2005. In March 2006, the advisory committees of FDA voted in favor of the return of natalizumab in the market as monotherapy in MS with a black box warning about PML. For this reason, it would be very important to have a reliable strategy to quantify the risk of PML in patients with MS [19]. Recently, Bloomgren et al. proposed a clinical flowchart based on three different risk factors, all associated to an increased risk of PML: positive status with respect to anti-JC virus antibodies, prior use of immunosuppressants and increasing duration of natalizumab treatment (prolonged natalizumab treatment likely increased PML risk, but some studies found no evidence of JCV reactivation in natalizumab-treated MS patients of 18 month follow-up study). The reason of this delay is not well characterized [23, 24]. Although important, these kind of studies are far from conclusive especially considering that up to 65% of healthy patients are seropositive to JCV. Other parameters are therefore necessary for a better risk stratification. Recently, a possible risk stratification based on the level of neutralizing activity of the anti-JCV humoral response has been proposed [25]. Overall, a better comprehension of the physiopathology of JCV and of PML would surely help in the identification of a panel of risk factors for a better stratification profile.

## 2. The Virus: JC Virus

### 2.1. Viral Genome and Structure

JCV is a member of the Polyomaviridae family, *Orthopolyomavirus* genus [26]. JCV and BK virus ((BKV), which causes a severe nephropathy in kidney transplant recipients) were the first two human polyomaviruses identified, both detected in 1971 [27]. JCV, like all polyomaviruses, is a nonenveloped, icosahedral, and small (*≈*40 nm) virus with a closed circular double stranded DNA genome [28]. The genome is approximately 5130 bp-long, although single isolate can be differ in length, due to alteration in their noncoding regions [29]. The genome can be divided in three different parts: (i) a noncoding control region (NCCR), (ii) early coding region sequences that are transcribed counterclockwise from NCCR, and (iii) late coding region sequences that are transcribed clockwise from NCCR [27]. 

The NCCR lies between the early and late coding regions and contains the origin of replication, the TATA box, the T antigen binding sites, the cellular transcription factor-binding sites, a bidirectional promoter, and an enhancer for transcription of early and late genes. NCCR is thought to be the main determinant of the viral tropism. Importantly, modifications on the NCCR region are associated with an increase of viral transcription and replication in patients with PML [30–32].

The early coding region spans 2.4 kb, and it encodes five proteins: the large T antigen, the small t antigen, and three different splice variants [33]. The large T antigen is a 688 amino acids nonstructural, multifunctional protein that regulates the switch from early to late viral proteins transcription and the replication of the viral genome. This protein interacts with a number of cellular proteins (such as retinoblastoma protein and p53), and its role in cancerogenesis is being investigated [34]. The small t antigen and the other T antigen variants are produced by cellular splicing of the large T antigen RNA, and these proteins perform multiple functions and may contribute to PML progression [35].

**Figure 2:**
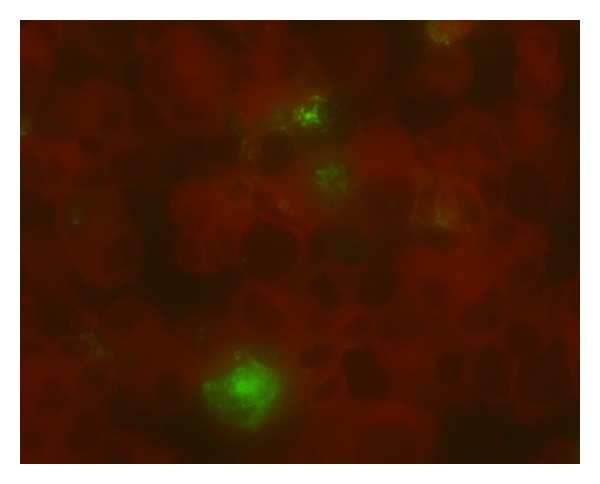
Immunofluorescence staining of COS7 infected by JCV (Mad4), five day after infection. The cells were stained with anti-VP1 monoclonal antibody (green-stained cells) and counterstained with Evans blue (red-stained cells).

The late region spans 2.3 kb and encodes four different proteins: agnoprotein, VP1, VP2, and VP3 (Table 1). The smallest protein is the agnoprotein, and it has been proposed to harbor functions during the late phase of infection as follows: interacts with large T antigen to control viral DNA replication and acts as a viroporin facilitating viral release from cells [36, 37]. The other three proteins are structural proteins: VP1 is the major capsid protein and allows the binding and entry into target cells; the VP2 and VP3 are assumed to function during escape from membranous structures and viral nuclear entry as described in SV40 [38–41]. JCV capsids are predicted to contain 360 molecules of VP1 arranged in 72 pentameric subunit, with each VP1 pentamer associated to a single VP2 or VP3 molecule to form the individual capsomeres [42] (Figure 2). Sequencing studies on VP1 from PML patients have shown characteristic mutations not evidenced in JCV isolates from healthy subjects. In particular, the mutations in positions L55, K60, N265, S267, and S269 are all limited to isolates from PML patients and cluster in close proximity to the receptor binding site. According to various authors, some of these mutations could alter the binding specificity of the virus from that dependent on sialic acid to that specific to other sugar moiety [43, 44]. PML-specific mutations are characteristic, but these are not present in all isolates from PML (statistical analysis of JCV sequences demonstrate, that 52% of PML patients carry JCV with one of these mutation, in VP1), and this suggests that VP1 mutations are not the only possible mechanisms leading to PML development.

Furthermore, several JCV VP1 loop-specific polymorphisms (restricted to four positions 74, 75, 117, and 128) have been described to be associated with favorable prognosis for PML [45]. 

### 2.2. Viral Lifecycle

The replicative cycle of JCV can be divided into two phases: early and late stages. The early stage begins with the initial interaction of the viral protein, VP1, with the surface of the host cell and continues until the onset of viral DNA replication. The late phase includes all the events that lead to the release of viral progeny. In both stages, viral and host proteins are essential for the complete viral lifecycle.

The early stage is initiated by adsorption of the virions to the cells surface. *In vitro* studies have demonstrated that JCV requires N-linked glycoproteins containing terminal *α*(2,6)-linked sialic acid to successfully bind human cells [46]. Recently, LSTc pentasaccharide has been described as functional receptors motif for this interaction [47]. Other studies have also evidenced the importance of the serotonin receptor 5HT_2A_ (5HT_2A_R) in viral entry [48]. However it is important to remember that JCV is able to bind different cell lines without producing viral progeny, evidencing the importance of intracellular cell type-specific factors in determining permissiveness to JCV replication [49, 50]. For these reasons, the tropism of JCV turns out to be very narrow; in fact, the virus productively infects stromal cells in tonsillar tissues, some B cells, CD34+ hematopoietic cells, oligodendrocytes, and astrocytes of the human brain.

 Similarly, to other DNA viruses, JCV penetrates into the cytoplasm by clathrin-dependent endocytosis [51, 52]. Once inside the cell, JCV is transported through the cytosol to the nucleus by the endosomes, traffics to the endoplasmatic reticulum, and subsequently binds to nuclear pore complexes. The nucleus is the site of viral replication and viral assembly. As with the other members of the family, JCV lifecycle exhibits a fine temporal regulation and is particularly slow (DNA replication is undetectable for some days) [53]. Initially, upon entering the nucleus, transcription of the early viral genes occurs (large T and small t antigens, proteins required for the viral DNA replication), followed by viral DNA replication. After the complete DNA replication, the late viral genes (VP1, VP2, VP3, and agnoprotein) are transcribed [27]. Transcription is regulated by cell-specific factors, while DNA replication is most likely regulated by species-specific factors, and for this reason, JCV has a limited host replication tropism; thus, *in vitro* cell transfection with JCV DNA results in the infection of only those cell types known to allow infection *in vivo* (as tonsillar stromal cells, B cells, and CD34+ hemopoietic cells). In particular, only certain cells have the necessary protein to allow complete viral lifecycle, for example, the nuclear transcription factor NF-1X has been described as a cell-specific regulator of JCV transcription, and this protein is expressed at higher concentration in human brain cells than in other human cells or in nonhuman brain cells [54]. 

Expression of the viral structural proteins leads to the assembly of the viral capsid. The newly packaged virion progeny is thought to be released by lysis of the host cell, although electron microscopy observations report secretion of virions from the plasma membrane of intact cells. It remains to be determined whether cell lysis or intracellular vesicular transport is the preferred pathway for the release of JCV progeny virions [55].

### 2.3. JCV Reactivation: The Clinical Picture and the Laboratory Diagnosis

After infecting the host, the virus can persist in at least two forms: nonpathogenic and a pathogenic forms. In particular, nonpathogenic form is most frequently found in urine, and its NCCR is not rearranged (named “archetype”), while the pathogenic form is principally detected in brain of PML patients, and its NCCR rearrangements (named “prototype”) include deletions and duplications of specific sequence elements (however, it is important to remember that postmortem studies have shown the presence of the prototype also in the brain of non-PML subjects) [56]. 

The pathogenesis of PML is due to the infection of oligodendrocytes by JCV [2, 3]. It is still widely discussed whether PML derives from JCV reactivation within oligodendrocytes infected during the initial phases of the infection or from its peripheral reactivation with a novel massive crossing of the BBB. As a consequence, oligodendrocytes undergo cytolytic destruction that results in loss of myelin, thus leading to the appearance of foci of demyelination, which initially are microscopic and asymmetrically distributed in space. As the disease progresses, the areas of demyelination enlarge and these foci may coalesce, making them visible on gross examination in cut sections of the brain. In addition to the oligodendroglial pathology, greatly hypertrophic giant pleomorphic astrocytes may be observed in areas of demyelination in 80% of cases [57]. The progression of the disease is usually very rapid and leads to death in less than one year from diagnosis, although it was observed that some PML patients can survive for many years [58].

Since PML involves the subcortical white matter, the lesions may manifest as a wide variety of neurological disturbances. The three characteristic symptoms at onset and during disease progression are (i) visual deficit, (ii) motor impairment, and (iii) change in mentation. The most common sign (35–45%) is visual deficit, while motor weakness is the initial sign in 25 to 33% of cases, and approximately one-third of patients shows a change in mentation, as personality change, difficulty with memory, emotional lability, and dementia. Other common symptoms are headache, vertigo, seizure, sensory deficit, parkinsonism, aphasia, and neglect syndrome [59, 60].

The expanding spectrum of iatrogenic conditions favouring PML and the frequent occurrence of atypical cases explain the importance of definitive clinic-radiological and laboratory diagnosis. Diagnostic tests investigating PML include neuroimaging, electroencephalography, component analysis of celebrospinal fluid (CSF), biopsy, and PCR. In a patient with PML, a computerized tomography (CT) scan shows nonenhancing, subcortical hypodensities, that correspond to areas of demyelination. Magnetic resonance imaging (MRI) is superior to CT scanning, in fact it shows not only the number but also the extent of the lesions. Electroencephalography (EEG) is both insensitive and nonspecific for PML, but it may corroborate the presence of a lesion seen on neuroimaging. In particular, EEG shows focal slowing corresponding to white matter lesions and generalized slowing with advancing disease. CSF findings are nonspecific, with most patients demonstrating a normal profile, but in some patients elevation in protein or an increased cell count could be present. All the above may give a strong suspicion of PML, but the confirmatory test is the demonstration of the presence of JCV DNA in CSF or brain by molecular methods [61]. In this test the specificity and the sensitivity are very important; in fact, the former has to be 100%, without cross-reaction with other polyomaviruses, and the later has to be able to detect even very few copies of viral DNA [62]. 

A problem that may be encountered with serological analysis is related to the high homology between polyomaviruses (e.g., BK virus display 75% homology with JCV) and in serum may be present cross-reactive antibodies that may give a false positive. Since about 65% of the healthy population is positive for JCV-specific antibodies, the fact that a patient does not present these antibodies could be considered as exclusion diagnosis for PML [27].

## 3. The Role of the Immune System: Friend or Foe?

### 3.1. Role of Immune System in Viral Pathway

The initial infection with JCV is thought to occur in tonsillar tissue after inhalation, although transmission of the virus through ingestion of contaminated food or water has also been suggested. Tonsillar lymphocytes infected with JCV carry virions to the kidney and bone marrow, the primary sites of viral latency; though several studies examined non-PML, normal brain tissue has suggested that the virus might enter or persist in the brain causing a latent infection that might reactivate in case of immune suppression, leading to a productive infection in oligodendrocytes. Another suggested model through which the lymphocytes may contribute to the dissemination of the infection is a sort of JCV association with the cell membrane without internalization; this could explain why viral DNA, but not RNA, is often detected in lymphocytes [49, 63]. 

The dissemination to the brain remains to be fully elucidated, even if a hematogenous route of infection of the CNS has been suggested, following a possible “Trojan horse” mechanism for the BBB crossing. This hypothesis is supported by the presence of infected B lymphocytes in multiple PML brain tissue samples [64]. Other studies have shown that JCV may also infect microvascular endothelial cells through infected lymphocytes, and thereby possibly cross the BBB as free virus [62, 64, 65].

**Figure 3:**
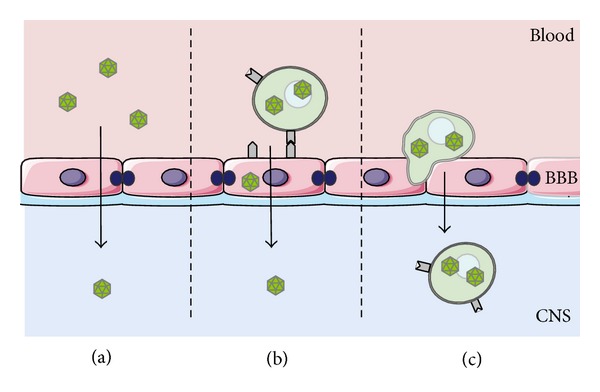
Mechanisms of PML pathogenesis. The necessary condition for the PML developments is that oligodendrocytes are infected by JCV. In this figure the three ways through which the virus could cross the BBB are represented: (a) as free virus or (b) the infection of the endothelial cells of the barrier by JCV-infected B cells (c) using the B cells as “a Trojan horse.”

Once JCV, as free virus or associated to B cells, crosses the BBB (Figure 3), at least three other events must occur so that the PML develops: (i) the host immune system must be compromised or altered, (ii) the viral NCCR must acquire changes that increase viral transcription and replication, and (iii) DNA binding factors that bind to recombined NCCR sequence motif must be present or upregulated in infected hematopoietic cells, B cells, or glial cells. Only when all these conditions are present, PML develops.

### 3.2. Immune Control of JCV

Although JCV is widespread in the population, only few people develop the disease, and this occurs only in the presence of underlying changes to the immune system. In fact, before the HIV pandemic and the introduction of immunomodulatory therapies, the PML was very rare, indicating that the infection is well controlled by the immune system. Epidemiological studies of PML in HIV-positive patients show that in pre-HAART era, the incidence of PML varied from 0.3 to 8%, but after the diffusion of antiviral treatment, its incidence decreased (from 0.7 cases/100 personyear to 0.07 cases/100 personyear) [66]. These data suggest that the cellular branch of the immune system probably plays the principal role in the control of the infection. In particular, in PML patients it was observed that the number and the functionality of CD4+ cells are reduced after stimulation with JCV antigen compared to normal control [67]. As a matter of fact, several studies have implicated an impairment of the T-cell response in the PML development, and in particular, an effective cytotoxic T lymphocyte response specific to the viral capsid protein has been associated with greater control of JCV and longer PML survival rates [68–71].

Moreover, a recent study shows differences in JCV-specific T-cell response during natalizumab treatment and in natalizumab-associated PML. In particular, it was observed that in patients treated with natalizumab, the magnitude and the quality of JCV-specific T-cell response did not change from the healthy patients, while in patients with natalizumab-associated PML, JCV-specific T cells were not measurable or JCV-specific T cells were dominated by IL-10 (human cytokine synthesis inhibitory factor) production, giving further evidence of the role of T-cell response in PML development [72].

On the contrary, the role of the humoral response has not been well defined yet. In fact, PML patients have substantial antibody titer direct against viral capsid protein before and during disease [73], and in particular it has been demonstrated that anti-JCV IgG are synthesized intrathecally but this was not associated with an improvement in their clinical outcome [74]. Also, the virus seems to have adapted to replicate and disseminate through B cells and their progenitors. However, when the human immune response against viruses that are causing persistent and latent infection has been dissected with modern tools allowing unprecedented accuracy [75, 76], it has been shown not only that the single antibody clones are endowed with a very different neutralizing activity [77–79] and that effective antibodies are very rare [80] but also that a part of the response can also have a biological activity not necessarily beneficial for the host [81–84]. Considering these aspects, it could be very important to better investigate this crucial aspect of the virus-host interplay, by identifying the role played by selected antibody clones capable of effectively neutralizing the viral particles, in case of peripheral reactivation. 

## 4. Treatment of PML: Old and New Strategies

### 4.1. Immune Reconstitution Inflammatory Syndrome (IRIS)

Since the etiopathological origin of PML is associated with compromised or altered host immune response, the immune reconstitution is actually considered the best strategy for an improved outcome. In particular, the immune reconstitution is based on the reduction of HIV load through HAART in the case of HIV-positive PML patients and on the elimination or reduction of the immunomodulatory drug in the case of immunomodulatory-treated PML patients [10, 85]. In this second group of patients, there are two strategies that can be adopted: the plasma exchange and the administration of intravenous immunoglobulins. In particular, plasma exchange has been safely and successfully used to eliminate free unbound natalizumab [86–88]. The second therapeutic option is the administration of intravenous immunoglobulins, in the hope that they may somehow limit the binding of the immunomodulatory drug to its target [89, 90]. 

However, the immune reconstitution may have very serious consequences; in fact it, can be associated with increasing inflammation and a clinical deterioration called immune reconstitution inflammatory syndrome (IRIS) [91]. In fact, when the immune system is reconstituted, fully functional and activated T cells regain access to the CNS compartment, initiating a strong inflammation within the brain, as a side effect of the massive destruction of virus-infected cells. IRIS is usually associated with an increasing CD4+ cell count and an exaggerated reaction of CD8+ T cells, especially in HIV-positive patients [92–95]. Inflammation can be visualized by contrast-enhancing lesions on MRI due to the open BBB. IRIS can lead to a rapid deterioration of the patient's clinical state and death in 30–50% of cases [96]. An effective therapeutic treatment for IRIS does not exist, even if an immunomodulatory therapy able to attenuate the T cells response is suggested [94].

### 4.2. Antiviral Drugs

Currently, no anti-JCV-specific drugs are available; a number of treatment options targeting different stages in the viral life cycle have been proposed.

The use of drugs potentially interfering with the viral entry has been suggested. A treatment for the inhibition of viral entry includes blocking access to 5HT_2A_R by antibodies or by serotonin receptor agonists (chlorpromazine and clozapine). Some authors described that monoclonal antibodies to 5HT_2A_R blocked infections of glial cells by JCV, while chlorpromazine inhibits clathrin-dependent endocytosis and in combination of clozapine can block the glial infection. However, these drugs have serious side effects and toxicity issues. Recently, newer antipsychotics (ziprasidone, risperidone, and olanzapine) have been shown to reduce JCV infection up to 10 fold in an *in vitro* system, but further studies are warranted to determine efficacy in PML treatment *in vivo*, either alone or in combination therapy with other drugs [51, 97].

 Many broad-spectrum nucleoside analog chemotherapeutics (including cytosine arabinoside, Ara-C, cidofovir, and CDV) that target DNA replication have been used to inhibit JCV replication without much success. CDV is an acyclic nucleotide phosphonate analog of deoxycytosine monophosphate; due to its inhibitory action on DNA polymerase, its effect on JCV has been tested with contradictory results. The low efficacy of CDV may reflect poor penetration, and severe side effects have been reported [98, 99]. Recently, a lipid derivative of CVD (a hexadecycloxypropyl lipid conjugate of CVD, called CMX001) was found to reduce JCV replication, with no significant toxicity in cell cultures derived from human fetal brain, suggesting that it could be a promising candidate for the treatment of PML [100]. Ara-C is another nucleoside analog, and it was effective in decreasing viral replication in cultured human neuroglial cells. Limitations of this drug could be its short half-life, poor ability to cross the BBB, and bone marrow toxicity [90].

Other drugs that inhibit viral DNA replication act on DNA topoisomerase. Topotecan blocked JCV DNA replication with no effect on host transcription and translation, but other studies should be conducted before using the drug in patients [101].

As described, all tested therapeutic options show no significant impact on survival or neurological improvement. The ineffectiveness of these molecules can be explained by many factors, such as the low ability to cross BBB, the variability of the viral structures, and the extreme complexity of the JCV/host interplay.

## 5. Discussion and Future Perspectives

In the literature, PML was considered a rare disease, and, before the HIV pandemic and the availability of immunomodulatory drugs, it was only associated with neoplasms impairing the immune system, such as chronic lymphocytic leukemia or Hodgkin's lymphoma. In the last two decades, the incidence of PML has begun to increase exponentially. In patients treated with immunomodulatory therapy, the incidence of PML depends on the drug used in therapy and on the treated disease. Of particular interest is the PML incidence in patients who receive natalizumab (approximately 1/500), a very effective drug mainly used in multiple sclerosis patients. This is a completely new scenario, especially if compared with HIV patients that were, in the past, almost irreversibly condemned to a death due to other, non-JCV-related, opportunistic infections. Indeed, although potentially at high risk, natalizumab is still being marketed for its high benefits in the MS treatment. These data lead to two very important considerations: the first regarding the management of the patient who is subjected to immunomodulatory therapy, and the second the treatment to be adopted in case of PML development.

The management of the patients is based on the identification of patients who truly are at high risk of developing the disease after immunomodulatory therapy. Recently, three risk factors have been proposed: presence in the serum of anti-JCV antibodies, prior use of immunosuppressants, and duration or natalizumab treatment. Unfortunately, this analysis shows limitations based primarily on the inadequacy of information on the pathophysiology of the disease and of the JCV biology. These are also the reasons why, to date, there is no specific and effective therapy for the treatment of PML. New therapies have been proposed and some of these appear to be very promising. In particular, many recent works have focused on the possible role that the main viral structural protein (VP1) involved in viral entry may have in the PML etiopathogenesis, suggesting it as a potential drug target. On the diagnostic side, it was recently described that mutations on VP1 are associated with PML, but it remains to be determined whether these changes influence PML risk. On the therapeutic side, a recent study by Balduzzi et al. [102] reported that the generation *in vitro* of JCV-specific CD8+ T cells using 15-mer peptides derived from VP1 and large T antigen and its clinical use in an allogenic hematopoietic stem cells recipients with PML may be important in the control of PML. Although not reported in the previously mentioned study, it is important to remember that the risk of IRIS is mainly related to an exaggerated CD8+ T cell response [95]. Furthermore, studies on the combination of low-dose chlorpromazine and neutralizing antibodies showed their possible use in prophylactic and therapeutic treatment of PML [103].

Other immunological paths are therefore to be considered and could not only allow a better comprehension of JCV-host interplay but, hopefully, also a better clinical management of PML patients, similarly to what has already happened for other persistent and latent viral infections. In particular, the clarification of the role played by the humoral response in controlling JCV dissemination to CNS and, more generally, a better understanding of the molecular features of this crucial aspect of the virus-host interplay can be crucial for opening new vistas in this field. It is intriguing that in the era of monoclonal antibody-related opportunistic infections, antiviral compound of the same class of drug could be potentially useful. In particular, the anti-JCV human humoral response should be dissected and studied. The possible activity of human neutralizing monoclonal antibodies directed against conserved regions of VP1 should be considered, as already happened for other viral infections where the role of the humoral response was for too long considered negligible or too limited in breadth [75, 80, 83, 104–110], since even if the overall contribution of the antibodies to the host-virus interplay can appear not too relevant, the role played by selected antibody subpopulations could all the same be of great importance. 

## Figures and Tables

**Table 1:** Nonstructural and structural viral protein.

Protein	Molecular weight	No. of amino acids	Function
Early protein (transcribed counterclockwise from NCCR)			
Large T antigen	79,305	688	Nonstructural protein. role in viral replication and transcription, interaction with host protein and probably in cancerogenesis
Small t antigen	20,236	172	Viral replication
Splice variants called T_135_′, T_136_′, and T_165_′			Viral DNA and cancerogenesis

Late protein (transcribed clockwise from NCCR)			
VP1	39,606	354	Major capsid protein, role in cellular binding and entry functions, and interaction with host receptors; it mediates hemagglutination
VP2	37,366	344	Minor capsid protein, assumed role on escape from membranous structures and nuclear import
VP3	25,743	225	Minor capsid protein assumed role on escape from membranous structures and nuclear import
Agnoprotein	8,081	71	The smaller protein that facilities capsid assembly. It is proposed as a viroporin
